# *NTHL1* is a recessive cancer susceptibility gene

**DOI:** 10.1038/s41598-023-47441-w

**Published:** 2023-11-30

**Authors:** Anna K. Nurmi, Liisa M. Pelttari, Johanna I. Kiiski, Sofia Khan, Mika Nurmikolu, Maija Suvanto, Niina Aho, Tiina Tasmuth, Eija Kalso, Johanna Schleutker, Anne Kallioniemi, Päivi Heikkilä, FinnGen, Aarno Palotie, Mark Daly, Bridget Riley-Gillis, Howard Jacob, Dirk Paul, Slavé Petrovski, Heiko Runz, Sally John, George Okafo, Nathan Lawless, Heli Salminen-Mankonen, Robert Plenge, Joseph Maranville, Mark McCarthy, Margaret G. Ehm, Kirsi Auro, Simonne Longerich, Anders Mälarstig, Katherine Klinger, Clement Chatelain, Matthias Gossel, Karol Estrada, Robert Graham, Robert Yang, Chris O’Donnell, Tomi P. Mäkelä, Jaakko Kaprio, Petri Virolainen, Antti Hakanen, Terhi Kilpi, Markus Perola, Jukka Partanen, Anne Pitkäranta, Taneli Raivio, Jani Tikkanen, Raisa Serpi, Tarja Laitinen, Veli-Matti Kosma, Jari Laukkanen, Marco Hautalahti, Outi Tuovila, Raimo Pakkanen, Jeffrey Waring, Fedik Rahimov, Ioanna Tachmazidou, Chia-Yen Chen, Zhihao Ding, Marc Jung, Shameek Biswas, Rion Pendergrass, David Pulford, Neha Raghavan, Adriana Huertas-Vazquez, Jae-Hoon Sul, Xinli Hu, Åsa Hedman, Manuel Rivas, Dawn Waterworth, Nicole Renaud, Ma’en Obeidat, Samuli Ripatti, Johanna Schleutker, Mikko Arvas, Olli Carpén, Reetta Hinttala, Johannes Kettunen, Arto Mannermaa, Katriina Aalto-Setälä, Mika Kähönen, Johanna Mäkelä, Reetta Kälviäinen, Valtteri Julkunen, Hilkka Soininen, Anne Remes, Mikko Hiltunen, Jukka Peltola, Minna Raivio, Pentti Tienari, Juha Rinne, Roosa Kallionpää, Juulia Partanen, Ali Abbasi, Adam Ziemann, Nizar Smaoui, Anne Lehtonen, Susan Eaton, Sanni Lahdenperä, Natalie Bowers, Edmond Teng, Fanli Xu, Laura Addis, John Eicher, Qingqin S. Li, Karen He, Ekaterina Khramtsova, Martti Färkkilä, Jukka Koskela, Sampsa Pikkarainen, Airi Jussila, Katri Kaukinen, Timo Blomster, Mikko Kiviniemi, Markku Voutilainen, Tim Lu, Linda McCarthy, Amy Hart, Meijian Guan, Jason Miller, Kirsi Kalpala, Melissa Miller, Kari Eklund, Antti Palomäki, Pia Isomäki, Laura Pirilä, Oili Kaipiainen-Seppänen, Johanna Huhtakangas, Nina Mars, Apinya Lertratanakul, Coralie Viollet, Marla Hochfeld, Jorge Esparza Gordillo, Fabiana Farias, Nan Bing, Margit Pelkonen, Paula Kauppi, Hannu Kankaanranta, Terttu Harju, Riitta Lahesmaa, Hubert Chen, Joanna Betts, Rajashree Mishra, Majd Mouded, Debby Ngo, Teemu Niiranen, Felix Vaura, Veikko Salomaa, Kaj Metsärinne, Jenni Aittokallio, Jussi Hernesniemi, Daniel Gordin, Juha Sinisalo, Marja-Riitta Taskinen, Tiinamaija Tuomi, Timo Hiltunen, Amanda Elliott, Mary Pat Reeve, Sanni Ruotsalainen, Audrey Chu, Dermot Reilly, Mike Mendelson, Jaakko Parkkinen, Tuomo Meretoja, Heikki Joensuu, Johanna Mattson, Eveliina Salminen, Annika Auranen, Peeter Karihtala, Päivi Auvinen, Klaus Elenius, Esa Pitkänen, Relja Popovic, Margarete Fabre, Jennifer Schutzman, Diptee Kulkarni, Alessandro Porello, Andrey Loboda, Heli Lehtonen, Stefan McDonough, Sauli Vuoti, Kai Kaarniranta, Joni A. Turunen, Terhi Ollila, Hannu Uusitalo, Juha Karjalainen, Mengzhen Liu, Stephanie Loomis, Erich Strauss, Hao Chen, Kaisa Tasanen, Laura Huilaja, Katariina Hannula-Jouppi, Teea Salmi, Sirkku Peltonen, Leena Koulu, David Choy, Ying Wu, Pirkko Pussinen, Aino Salminen, Tuula Salo, David Rice, Pekka Nieminen, Ulla Palotie, Maria Siponen, Liisa Suominen, Päivi Mäntylä, Ulvi Gursoy, Vuokko Anttonen, Kirsi Sipilä, Hannele Laivuori, Venla Kurra, Laura Kotaniemi-Talonen, Oskari Heikinheimo, Ilkka Kalliala, Lauri Aaltonen, Varpu Jokimaa, Marja Vääräsmäki, Outi Uimari, Laure Morin-Papunen, Maarit Niinimäki, Terhi Piltonen, Katja Kivinen, Elisabeth Widen, Taru Tukiainen, Niko Välimäki, Eija Laakkonen, Jaakko Tyrmi, Heidi Silven, Eeva Sliz, Riikka Arffman, Susanna Savukoski, Triin Laisk, Natalia Pujol, Janet Kumar, Iiris Hovatta, Erkki Isometsä, Hanna Ollila, Jaana Suvisaari, Thomas Damm Als, Antti Mäkitie, Argyro Bizaki-Vallaskangas, Sanna Toppila-Salmi, Tytti Willberg, Elmo Saarentaus, Antti Aarnisalo, Elisa Rahikkala, Kristiina Aittomäki, Fredrik Åberg, Mitja Kurki, Aki Havulinna, Juha Mehtonen, Priit Palta, Shabbeer Hassan, Pietro Della Briotta Parolo, Wei Zhou, Mutaamba Maasha, Susanna Lemmelä, Aoxing Liu, Arto Lehisto, Andrea Ganna, Vincent Llorens, Henrike Heyne, Joel Rämö, Rodos Rodosthenous, Satu Strausz, Tuula Palotie, Kimmo Palin, Javier Gracia-Tabuenca, Harri Siirtola, Tuomo Kiiskinen, Jiwoo Lee, Kristin Tsuo, Kati Kristiansson, Kati Hyvärinen, Jarmo Ritari, Katri Pylkäs, Minna Karjalainen, Tuomo Mantere, Eeva Kangasniemi, Sami Heikkinen, Nina Pitkänen, Samuel Lessard, Clément Chatelain, Lila Kallio, Tiina Wahlfors, Eero Punkka, Sanna Siltanen, Teijo Kuopio, Anu Jalanko, Huei-Yi Shen, Risto Kajanne, Mervi Aavikko, Helen Cooper, Denise Öller, Rasko Leinonen, Henna Palin, Malla-Maria Linna, Masahiro Kanai, Zhili Zheng, L. Elisa Lahtela, Mari Kaunisto, Elina Kilpeläinen, Timo P. Sipilä, Oluwaseun Alexander Dada, Awaisa Ghazal, Anastasia Kytölä, Rigbe Weldatsadik, Kati Donner, Anu Loukola, Päivi Laiho, Tuuli Sistonen, Essi Kaiharju, Markku Laukkanen, Elina Järvensivu, Sini Lähteenmäki, Lotta Männikkö, Regis Wong, Auli Toivola, Minna Brunfeldt, Hannele Mattsson, Sami Koskelainen, Tero Hiekkalinna, Teemu Paajanen, Kalle Pärn, Mart Kals, Shuang Luo, Shanmukha Sampath Padmanabhuni, Marianna Niemi, Mika Helminen, Tiina Luukkaala, Iida Vähätalo, Jyrki Tammerluoto, Sarah Smith, Tom Southerington, Petri Lehto, Kristiina Aittomäki, Carl Blomqvist, Heli Nevanlinna

**Affiliations:** 1 Department of Obstetrics and Gynecology, University of Helsinki and Helsinki University Hospital, Biomedicum Helsinki, P.O. Box 700, 00290 Helsinki, Finland; 2 Department of Anaesthesiology, Intensive Care and Pain Medicine, University of Helsinki and Helsinki University Hospital, Helsinki, Finland; 3 Institute of Biomedicine, University of Turku, and FICAN West Cancer Centre, and Department of Genomics, Laboratory Division, Turku University Hospital, Turku, Finland; 4 Tays Cancer Center, Tampere University Hospital, and BioMediTech Institute and Faculty of Medicine and Health Technology, Tampere University, and Fimlab Laboratories, Tampere, Finland; 5 Department of Pathology, University of Helsinki and Helsinki University Hospital, Helsinki, Finland; 6 Department of Clinical Genetics, University of Helsinki and Helsinki University Hospital, Helsinki, Finland; 7 Department of Oncology, University of Helsinki and Helsinki University Hospital, Helsinki, Finland; 8 Institute for Molecular Medicine Finland (FIMM), HiLIFE, University of Helsinki, Helsinki, Finland; 9 Broad Institute of MIT and Harvard, Cambridge, MA USA; 10 Massachusetts General Hospital, Boston, MA USA; 11 AbbVie, Chicago, IL USA; 12 AstraZeneca, Cambridge, UK; 13 Biogen, Cambridge, MA USA; 14 Boehringer Ingelheim, Ingelheim am Rhein, Germany; 15 Bristol Myers Squibb, New York, NY USA; 16 Genentech, San Francisco, CA USA; 17 GlaxoSmithKline, Collegeville, PA USA; 18 GlaxoSmithKline, Espoo, Finland; 19 Merck, Kenilworth, NJ USA; 20 Pfizer, New York, NY USA; 21 Translational Sciences, Sanofi R&D, Framingham, MA USA; 22 Maze Therapeutics, San Francisco, CA USA; 23 Janssen Biotech, Beerse, Belgium; 24 Novartis Institutes for BioMedical Research, Cambridge, MA USA; 25 HiLIFE, University of Helsinki, Helsinki, Finland; 26 Auria Biobank, University of Turku and Hospital District of Southwest Finland, Turku, Finland; 27 THL Biobank, Finnish Institute for Health and Welfare (THL), Helsinki, Finland; 28 Finnish Red Cross Blood Service and Finnish Hematology Registry and Clinical Biobank, Helsinki, Finland; 29 Helsinki Biobank, University of Helsinki and Hospital District of Helsinki and Uusimaa, Helsinki, Finland; 30 Northern Finland Biobank Borealis, University of Oulu and Northern Ostrobothnia Hospital District, Oulu, Finland; 31 Finnish Clinical Biobank Tampere, Tampere University and Pirkanmaa Hospital District, Tampere, Finland; 32 Biobank of Eastern Finland, University of Eastern Finland and Northern Savo Hospital District, Kuopio, Finland; 33 Central Finland Biobank, University of Jyväskylä and Central Finland Health Care District, Jyväskylä, Finland; 34 FINBB, Finnish Biobank Cooperative, Helsinki, Finland; 35 Business Finland, Helsinki, Finland; 36 GlaxoSmithKline, Stevenage, UK; 37 University of Stanford, Stanford, CA USA; 38 Janssen Research & Development, LLC, Spring House, PA USA; 39 Faculty of Medicine and Health Technology, Tampere University, Tampere, Finland; 40 Northern Savo Hospital District, Kuopio, Finland; 41 Northern Ostrobothnia Hospital District, Oulu, Finland; 42 University of Eastern Finland, Kuopio, Finland; 43 Pirkanmaa Hospital District, Tampere, Finland; 44 Hospital District of Helsinki and Uusimaa, Helsinki, Finland; 45 Hospital District of Southwest Finland, Turku, Finland; 46 GlaxoSmithKline, Brentford, UK; 47 Janssen Research & Development, LLC, Titusville, NJ USA; 48 Tampere University, Tampere, Finland; 49 Seinäjoki Central Hospital, Seinäjoki, Finland; 50 University of Gothenburg, Gothenburg, Sweden; 51 Novartis, Basel, Switzerland; 52 Finnish Institute for Health and Welfare (THL), Helsinki, Finland; 53 Janssen Research & Development, LLC, Boston, MA USA; 54 Novartis, Boston, MA USA; 55 Janssen-Cilag Oy, Espoo, Finland; 56 Department of Molecular Genetics, University of Lodz, Lodz, Poland; 57 Helsinki University Hospital and University of Helsinki, Helsinki, Finland; 58 Eye Genetics Group, Folkhälsan Research Center, Helsinki, Finland; 59 Research Unit of Oral Health Sciences, Faculty of Medicine, University of Oulu, Oulu, Finland; 60 Medical Research Center Oulu, Oulu University Hospital and University of Oulu, Oulu, Finland; 61 University of Helsinki, Helsinki, Finland; 62 University of Jyväskylä, Jyväskylä, Finland; 63 University of Oulu, Oulu, Finland; 64 Estonian Biobank, Tartu, Estonia; 65 Aarhus University, Aarhus, Denmark; 66 Department of Otorhinolaryngology-Head and Neck Surgery, University of Helsinki and Helsinki University Hospital, Helsinki, Finland; 67 Department of Medical Genetics, Helsinki University Central Hospital, Helsinki, Finland; 68 Transplantation and Liver Surgery Clinic, Helsinki University Hospital and University of Helsinki, Helsinki, Finland; 69 Finnish Red Cross Blood Service, Helsinki, Finland; 70 European Molecular Biology Laboratory, European Bioinformatics Institute, Cambridge, UK

**Keywords:** Cancer, Genetics

## Abstract

In search of novel breast cancer (BC) risk variants, we performed a whole-exome sequencing and variant analysis of 69 Finnish BC patients as well as analysed loss-of-function variants identified in DNA repair genes in the Finns from the Genome Aggregation Database. Additionally, we carried out a validation study of *SERPINA3* c.918-1G>C, recently suggested for BC predisposition. We estimated the frequencies of 41 rare candidate variants in 38 genes by genotyping them in 2482–4101 BC patients and in 1273–3985 controls. We further evaluated all coding variants in the candidate genes in a dataset of 18,786 BC patients and 182,927 controls from FinnGen. None of the variants associated significantly with cancer risk in the primary BC series; however, in the FinnGen data, *NTHL1* c.244C>T p.(Gln82Ter) associated with BC with a high risk for homozygous (OR = 44.7 [95% CI 6.90–290], P = 6.7 × 10^–5^) and a low risk for heterozygous women (OR = 1.39 [1.18–1.64], P = 7.8 × 10^–5^). Furthermore, the results suggested a high risk of colorectal, urinary tract, and basal-cell skin cancer for homozygous individuals, supporting *NTHL1* as a recessive multi-tumour susceptibility gene. No significant association with BC risk was detected for *SERPINA3* or any other evaluated gene.

## Introduction

Cancer is a genetic disease in which accumulating pathogenic variants give growth advantage to malignant cells. Eukaryotic cells have specialized pathways for the repair of different mutation types and others that control the cell cycle checkpoints or initiate apoptosis. Defective DNA damage response mechanisms increase genomic instability and may lead to tumour development^1^.

The validated breast cancer (BC) risk genes to date function primarily in DNA double-strand break and interstrand crosslink repair via the homologous recombination and the Fanconi anaemia (FA) pathways and in DNA damage checkpoint signalling^2,3^. The high-penetrance BC risk genes, *BRCA1* and *BRCA2*, encode proteins at the core of the pathways, promoting DNA repair in response to damage signalling^2^. The validated moderate-to-high risk BC predisposition genes, *PALB2*, *CHEK2*, *ATM*, *BARD1*, *RAD51C*, and *RAD51D*, have functions linked to *BRCA1* and *BRCA2*^2,3^. Studies on hereditary BC risk have most often focused on the DNA damage response genes. Other pathways may also be involved in the BC risk predisposition; for example, the syndromic cancer genes and the low-penetrance variants associated with BC risk show a wide range of affected pathways^2–4^.

The high- and moderate-risk variants in the established BC predisposition genes have an autosomal dominant inheritance pattern, even if with incomplete penetrance. Recessive model has also been suggested for increased risk of BC^5^, but to date, no recessive high- or moderate-risk BC susceptibility gene has been validated. Recently, several BC patients with pathogenic biallelic *NTHL1* variants have been described^6–12^, indicating recessive BC predisposition. Pathogenic variants in the *NTHL1* gene have been determined to cause a recessive multi-tumour syndrome, which is characterized especially by adenomatous polyposis and colorectal cancer (CRC), and with accumulating evidence, BC in women^6–13^.

The genes and causal variants contributing to a large proportion of the hereditary BC risk are yet to be discovered^4^. The genetic bottleneck events in the Finnish population have resulted in less overall variation and a higher frequency of loss-of-function (LoF) variants, including recessive disease variants, in the Finns compared to other Europeans^14,15^. This founder effect present in the Finns is advantageous for genetic research as it facilitates the detection of novel disease genes and variants. Only a few recurrent variants account for most of the pathogenic burden in the validated BC risk genes in Finnish BC patients^16^. High-risk *BRCA1/2* variants have been identified in about 21% of Finnish BC families and 1.8% of unselected BC patients^16–18^. The combined frequency of pathogenic variants in the other validated high- and moderate-risk BC susceptibility genes is about 10% in Finnish BC families and 5% in unselected BC patients^16^.

With the aim of identifying novel BC risk variants, we have performed a whole-exome sequencing (WES) and variant analysis of 69 patients from Finnish BC families as well as an analysis of predicted loss-of-function (pLoF) variants in 520 DNA repair genes, detected in approximately 11,000 Finns from the Genome Aggregation Database (gnomAD), and selected candidate risk variants for a case–control study. Additionally, a recent Finnish study reported a putative novel moderate-risk BC susceptibility variant *SERPINA3* c.918-1G>C^19^, warranting further validation. Here, we evaluated *SERPINA3* c.918-1G>C alongside the other candidate variants for BC risk.

## Results

**Figure 1:**
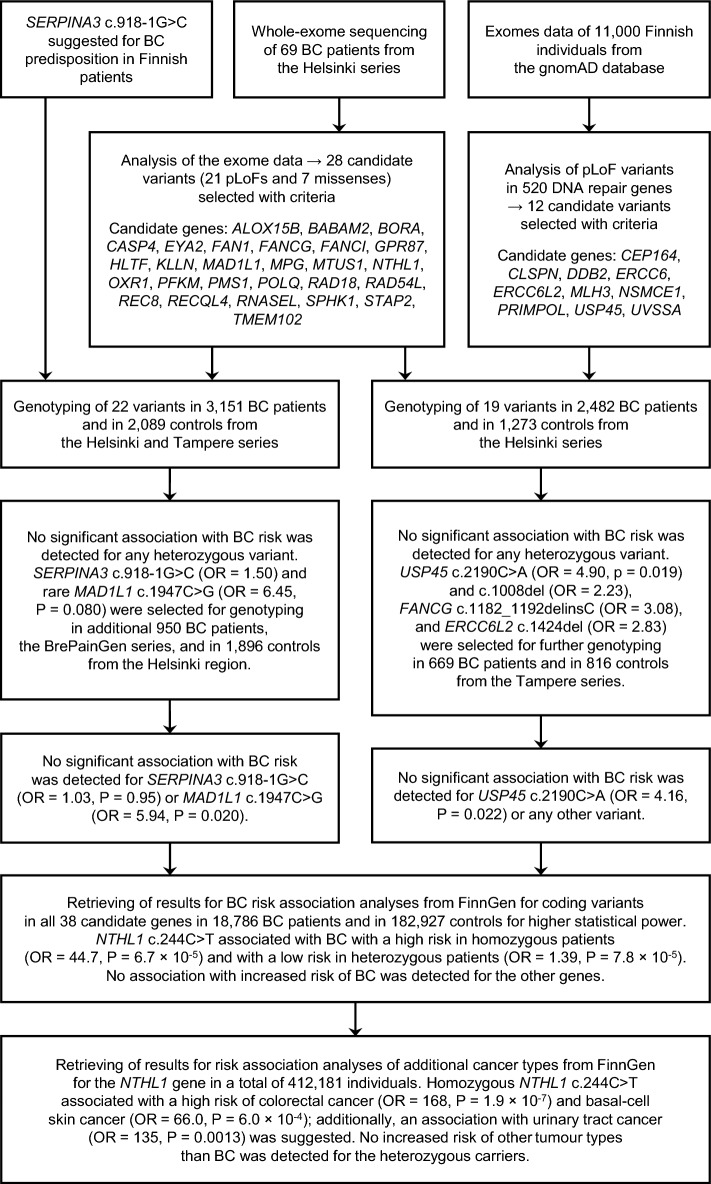
An overview of the work process and findings of the study.

We selected altogether 41 candidate variants in 38 genes, presented in detail in the Supplementary Table S1, for genotyping in BC patients and controls from the Helsinki and Tampere regions in Southern Finland and assessed the variants for cancer risk (Fig. 1). Finally, we retrieved the data for cancer risk association analyses from the FinnGen project and examined the candidate genes and variants in this large series of cancer patients and controls.

### Breast cancer risk association analyses in the Helsinki and Tampere series

We genotyped 19 of the selected candidate variants in 2482 BC patients and 1273 controls, 20 of the variants in 3151 BC patients and 2089 controls, and two of the variants in 4101 BC patients and 3985 controls from the Helsinki and Tampere regions. After the Bonferroni correction for multiple comparisons (P < 0.0012), none of the studied variants associated significantly with BC risk in this primary study (Table 1, Supplementary Table S2). We detected two variants, *MAD1L1* NM_001013836.2:c.1947C>G p.(Tyr649Ter) and *USP45* NM_001346022.3:c.2190C>A p.(Tyr730Ter), with a higher frequency in the patients than in the controls on a nominally significant level (P < 0.05) (Table 1); however, another pLoF in the *USP45* gene, NM_001346022.3:c.1008del p.(Val337SerfsTer9), was found only slightly more often in the patients than in the controls.

**Table 1:** Variant frequencies in breast cancer patients from the Helsinki and Tampere regions.

Variant	Group	Total	Carriers	%	OR	95% CI	P value
*MAD1L1*	Controls	3974	2	0.05			
c.1947C>G	All BC	4083	12	0.3	5.94	1.62–38.2	0.020
p.(Tyr649Ter)	*Familial BC*	1524	6	0.4	8.13	1.86–55.7	0.011
rs121908981	*Unselected BC*	3327	8	0.2	4.81	1.20–31.9	0.047
	ER-positive BC	3172	10	0.3	6.46	1.70–42.1	0.016
	ER-negative BC	720	2	0.3	5.61	0.67–46.9	0.085
*SERPINA3*	Controls	3978	10	0.3			
c.918-1G>C	All BC	4095	11	0.3	1.03	0.43–2.47	0.95
rs199710314	*Familial BC*	1527	3	0.2	0.75	0.17–2.46	0.66
	*Unselected BC*	3339	9	0.3	1.07	0.42–2.65	0.89
	ER-positive BC	3184	9	0.3	1.08	0.43–2.69	0.86
	ER-negative BC	720	1	0.1	0.54	0.03–2.83	0.56
*ERCC6L2*	Controls	2083	12	0.6			
c.1424del	All BC	3142	12	0.4	0.76	0.33–1.75	0.51
p.(Ile475ThrfsTer36)	*Familial BC*	1369	6	0.4	1.00	0.34–2.68	1.00
rs768081343	*Unselected BC*	2386	8	0.3	0.64	0.25–1.57	0.34
	ER-positive BC	2386	10	0.4	0.86	0.35–2.03	0.72
	ER-negative BC	573	1	0.2	0.42	0.02–2.20	0.41
*FANCG*	Controls	2086	1	0.05			
c.1182_1192delinsC	All BC	3147	6	0.2	3.08	0.53–58.2	0.30
p.(Glu395TrpfsTer5)	*Familial BC*	1368	4	0.3	4.49	0.66–87.9	0.18
rs397507559	*Unselected BC*	2391	3	0.1	2.21	0.28–44.8	0.49
	ER-positive BC	2389	5	0.2	3.39	0.55–65.0	0.27
	ER-negative BC	576	1	0.2	2.82	0.11–71.4	0.46
*NTHL1*	Controls	2081	17	0.8			
c.244C>T	All BC	3117	30	1.0	1.35	0.74–2.54	0.33
p.(Gln82Ter)	*Familial BC*	1357	16	1.2	1.77	0.86–3.64	0.12
rs150766139	*Unselected BC*	2370	22	0.9	1.25	0.66–2.41	0.49
	ER-positive BC	2366	17	0.7	0.99	0.49–1.99	0.98
	ER-negative BC	573	9	1.6	2.32	0.97–5.21	0.048
*USP45*	Controls	2088	3	0.1			
c.2190C>A	All BC	3148	21	0.7	4.16	1.42–17.7	0.022
p.(Tyr730Ter)	*Familial BC*	1368	12	0.9	5.49	1.70–24.6	0.0097
rs118066385	*Unselected BC*	2392	17	0.7	4.59	1.53–19.7	0.015
	ER-positive BC	2389	17	0.7	4.31	1.43–18.6	0.021
	ER-negative BC	576	2	0.3	2.59	0.33–16.2	0.31
*USP45*	Controls	2086	6	0.3			
c.1008del	All BC	3148	13	0.4	1.29	0.50–3.73	0.61
p.(Val337SerfsTer9)	*Familial BC*	1369	8	0.6	1.92	0.65–6.01	0.24
rs554927779	*Unselected BC*	2392	9	0.4	1.23	0.44–3.69	0.70
	ER-positive BC	2390	11	0.5	1.46	0.55–4.30	0.46
	ER-negative BC	575	2	0.3	1.24	0.18–5.53	0.80

The familial and the unselected patient groups overlap: 775 patients were included in both groups in the analyses of the *MAD1L1* and *SERPINA3* variants and 614 in the analyses of the other variants. Two of the *NTHL1* c.244C>T carriers included in the analysis were homozygous.

*FANCG* NM_004629.2:c.1182_1192delinsC p.(Glu395TrpfsTer5), *NTHL1* NM_002528.7:c.244C > T p.(Gln82Ter) (also known as NM_002528.6:c.268C > T p.(Gln90Ter) in reference to the previous transcript version), and *ERCC6L2* NM_020207.7:c.1424del p.(Ile475ThrfsTer36) (previously denoted as NM_020207.5:c.1457del p.(Ile486ThrfsTer36)) have been identified to cause recessive hereditary diseases with increased risk of cancer^6,9,20–23^. Here, we detected no significant association between the heterozygous pLoFs and BC risk. Of note, *FANCG* c.1182_1192delinsC was very rare in our patient series and only detected in 0.2% (6/3147) of the patients and in 0.05% (1/2086) of the controls. Only two patients were homozygous for the *NTHL1* c.244C > T variant, and we were unable to study any recessive BC risk associated with *NTHL1* in our patient series. No study subject was homozygous for *ERCC6L2* c.1424del.

We found *SERPINA3* NM_001085.5:c.918-1G>C with a similar frequency in the patients and in the controls and detected no association between the variant and BC risk. Previously, the c.918-1G>C carriers were reported to have a medullary breast tumour type more often than noncarriers^19^. Here, no c.918-1G>C carrier had medullary BC: eight patients had ductal, one patient had lobular, and two patients had carcinomas of mixed type.

The other studied variants were either detected only in a few patients or the analyses did not suggest an increased risk of BC (Supplementary Table S2).

### Breast cancer risk association analyses from FinnGen

To further evaluate the candidate genes and variants in a dataset with higher statistical power, we retrieved the results for BC risk association analyses from the FinnGen study, data release 10, for all coding variants in the studied genes in 18,786 Finnish BC patients and in 182,927 controls^15,24^. The FinnGen data also provided recessive association analyses for *NTHL1* c.244C>T and *ERCC6L2* c.1424del, which we were unable to perform in the Helsinki and Tampere BC series.

The genotype data suggested a low increased risk of BC for heterozygous *NTHL1* c.244C>T carriers in the additive model (odds ratio (OR) = 1.39 [95% confidence interval (CI) 1.18–1.64], P = 7.8 × 10^–5^) (Tables 2, 3). Carriers were detected with a similar frequency in the oestrogen receptor (ER)-positive patient group (OR = 1.41 [1.14–1.73], P = 0.0012) and in the ER-negative patient group (OR = 1.44 [1.06–1.95], P = 0.020) (Table 3). The recessive model suggested a notable risk of BC for homozygous individuals (OR = 44.7 [6.90–290], P = 6.7 × 10^–5^), both in the ER-positive patient group (OR = 82.1 [10.2–660], P = 3.4 × 10^–5^) and in the ER-negative patient group (OR = 86.3 [4.89–1523], P = 0.0023) (Table 3). Another, a much rarer pLoF in the *NTHL1* gene, c.674dup p.(Ser226ValfsTer39), was found only in heterozygous state (OR = 3.01 [0.67–13.6], P = 0.15) (Table 2); therefore, recessive analysis was not available for this variant.

**Table 2:** Breast cancer risk association analyses from FinnGen for heterozygous pLoF variants in the candidate genes.

Gene	Variant	Effect allele frequecy	OR	95% CI	P value
*ERCC6L2*	c.123dup p.(Ile42TyrfsTer5)	8.08 × 10^–5^	5.08	1.56–16.5	0.0070
*ERCC6L2*	c.1125dup p.(Ile376TyrfsTer7)	4.30 × 10^–5^	1.07	0.19–6.06	0.94
*ERCC6L2*	c.1424del p.(Ile475ThrfsTer36)^a^	3.78 × 10^–3^	1.09	0.89–1.33	0.42
*ERCC6L2*	c.1930C>T p.(Arg644Ter)	1.55 × 10^–4^	0.69	0.29–1.65	0.40
*FANCG*	c.832dup p.(Ala278GlyfsTer11)	1.10 × 10^–4^	0.84	0.26–2.74	0.78
*FANCG*	c.1076+1G>A	4.00 × 10^–5^	1.45	0.23–9.18	0.69
*MAD1L1*	c.150+1G>T	4.61 × 10^–5^	0.33	0.04–2.57	0.29
*MAD1L1*	c.538dup p.(Val180GlyfsTer47)	5.91 × 10^–5^	0.48	0.11–2.12	0.33
*MAD1L1*	c.1396C>T p.(Gln466Ter)	6.15 × 10^–5^	1.09	0.21–5.53	0.92
*MAD1L1*	c.1505+2T>A	1.54 × 10^–5^	3.73	0.40–34.8	0.25
*MAD1L1*	c.1947C>G p.(Tyr649Ter)^a^	9.47 × 10^–4^	0.87	0.59–1.27	0.47
*NTHL1*	c.244C>T p.(Gln82Ter)^a^	4.65 × 10^–3^	1.39	1.18–1.64	7.8 × 10^–5^
*NTHL1*	c.674dup p.(Ser226ValfsTer39)	6.15 × 10^–5^	3.01	0.67–13.6	0.15
*SERPINA3*	c.511C>T p.(Gln171Ter)	2.49 × 10^–4^	1.03	0.49–2.14	0.95
*SERPINA3*	c.918-1G>C^a^	1.96 × 10^–3^	1.15	0.86–1.54	0.35
*USP45*	c.7del p.(Val3Ter)	1.51 × 10^–4^	0.62	0.23–1.66	0.34
*USP45*	c.658G>T p.(Glu220Ter)	8.92 × 10^–5^	1.97	0.67–5.77	0.22
*USP45*	c.845+2T>C	4.28 × 10^–3^	0.86	0.71–1.03	0.10
*USP45*	c.1008del p.(Val337SerfsTer9)^a^	4.43 × 10^–4^	0.78	0.41–1.46	0.43
*USP45*	c.2190C>A p.(Tyr730Ter)^a^	1.70 × 10^–3^	0.90	0.67–1.21	0.48

The variants denoted with ^a^ were genotyped in the Helsinki and Tampere BC series. Reference transcripts: *ERCC6L2* NM_020207.7, *FANCG* NM_004629.2, *MAD1L1* NM_001013836.2, *NTHL1* NM_002528.7, *SERPINA3* NM_001085.5, and *USP45* NM_001346022.3.

**Table 3:** Cancer risk association analyses from FinnGen for the *NTHL1* c.244C>T variant.

Cancer type		Total number of individuals		Recessive model			Additive model		
		Patients	Controls	OR	95% CI	P value	OR	95% CI	P value
Breast	Breast cancer	18,786	182,927	44.7	6.90–290	6.7 × 10^–5^	1.39	1.18–1.64	7.8 × 10^–5^
	ER-positive breast cancer	10,404	182,678	82.1	10.2–660	3.4 × 10^–5^	1.41	1.14–1.73	0.0012
	ER-negative breast cancer	6188	182,678	86.3	4.89–1523	0.0023	1.44	1.06–1.95	0.020
Colon	Colorectal cancer	6847	314,193	168	24.4–1152	1.9 × 10^–7^	1.14	0.86–1.52	0.35
	Colorectal adenocarcinoma	5610	314,193	204	22.7–1837	2.1 × 10^–6^	0.99	0.73–1.36	0.96
	Colon cancer	4143	314,193	166	14.8–1856	3.4 × 10^–5^	1.19	0.83–1.70	0.35
	Colon adenocarcinoma	3212	314,193	224	10.8–4643	4.7 × 10^–4^	0.99	0.65–1.49	0.95
	Rectal cancer	2490	314,193	447	49.7–4023	5.2 × 10^–8^	1.04	0.67–1.63	0.85
	Adenocarcinoma, papilloma adenocarcinoma, and mucinous carcinoma of rectum	2545	314,193	472	52.1–4279	4.4 × 10^–8^	1.13	0.72–1.75	0.60
Urinary tract	Cancer of the urinary organs	2619	314,193	135	6.73–2713	0.0013	0.94	0.60–1.47	0.79
	Cancer of the renal pelvis	138	314,193	146	3.55–5985	0.0086	3.14	0.41–24.0	0.27
	Bladder cancer	2193	314,193	238	8.92–6334	0.0011	1.45	0.87–2.39	0.15
Other	Basal-cell carcinoma of the skin	20,506	314,193	66.0	6.02–723	6.0 × 10^–4^	1.16	0.97–1.38	0.11
	Prostate cancer	15,199	131,266	365	1.97–67,342	0.027	1.04	0.84–1.29	0.73

The controls included only women for BC and only men for prostate cancer. The risk association analyses of other cancer types for heterozygous *NTHL1* c.244C>T carriers are presented in the Supplementary Table S4.

No variant significantly associated with BC risk (P < 0.0012) in the other candidate genes (Table 2, Supplementary Table S3). In more detail, no risk association was detected for *MAD1L1* c.1947C>G (OR = 0.87 [0.59–1.27], P = 0.47), *SERPINA3* c.918-1G>C (OR = 1.15 [0.86–1.54], P = 0.35), or *USP45* c.2190C>A (OR = 0.90 [0.67–1.21, P = 0.48). *FANCG* c.1182_1192delinsC was not included in the FinnGen data, but two other, albeit very rare *FANCG* pLoFs, c.832dup p.(Ala278GlyfsTer11) and c.1076+1G>A, were detected in the study subjects. *ERCC6L2* c.1424del was found with a similar frequency in the patients as in the controls (OR = 1.09 [0.89–1.33], P = 0.42); however, another pLoF in *ERCC6L2*, c.123dup p.(Ile42TyrfsTer5), was more frequent in the patients compared with the controls (OR = 5.08 [1.56–16.5], P = 0.0070). Of the *ERCC6L2* variants, recessive analysis was available only for c.1424del (recessive OR = 20.6 [1.40–303], P = 0.027).

### Breast tumour characteristics of the patients with the *NTHL1* c.244C>T variant

We were able to evaluate the breast tumours of the patients with the *NTHL1* c.244C>T variant further in the Helsinki and Tampere BC series. Two patients from Helsinki were homozygous for the variant. One homozygous patient had been diagnosed with BC at the age of 41 years and with rectal and cecum cancers at the age of 47 years. The breast tumour of this patient was ER-positive and progesterone receptor (PR)-positive ductal carcinoma with grade 3. The other homozygous patient had BC at the age of 47 years and cancer of the sigmoid colon at the age of 51 years. This patient had an ER-positive, PR-positive, and HER2-negative ductal breast carcinoma with grade 2. Neither of the homozygous patients had a family history of BC or OC.

The average age of BC diagnosis among the 28 heterozygous carriers was 58.3 years (range 39–88 years), which was higher than the average age of 56.5 years (range 21–95) for all patients in the Helsinki and Tampere series. Of the heterozygous carriers, 75.0% (21/28) had ductal, 17.9% (5/28) had lobular, and 7.1% (2/28) had other invasive breast tumour type. Additionally, 65.4% (17/26) of the patients had ER-positive and 34.6% (9/26) had ER-negative BC, including three patients with triple-negative BC, and 78.3% (18/23) of the patients had a breast tumour with a grade 2 or 3. Additional cancer diagnoses were available only for the patients from the Helsinki BC series: of the 18 heterozygous carriers, two patients had bilateral BC, one had BC and uterus cancer, and one had BC and pancreatic cancer. One patient with bilateral BC and one other heterozygous BC patient also carried a pathogenic *CHEK2* c.1100del variant; no other high- or moderate-risk BC predisposition variants had been found in the *NTHL1* c.244C>T carriers from Helsinki.

### Association of *NTHL1* c.244C>T with increased risk of other cancer types than breast cancer

We obtained the data for recessive risk association analyses from FinnGen for all malignant tumour types diagnosed in the individuals homozygous for the *NTHL1* c.244C>T variant. Besides BC, homozygous *NTHL1* c.244C>T significantly associated with a high risk of CRC (OR = 168 [24.4–1152], P = 1.9 × 10^–7^) and basal-cell skin cancer (OR = 66.0 [6.02–723], P = 6.0 × 10^–4^) (Table 3). Additionally, the results suggested an increased risk of urinary tract cancers (OR = 135 [6.73–2713], P = 0.0013).

Ten individuals with the homozygous *NTHL1* c.244C>T variant were identified in the FinnGen study: nine of them had been diagnosed with one or multiple tumour types as verified by the Finnish Cancer Registry, and one had no cancer diagnosis. The diagnosed malignant tumour types were rectal, colon, breast, bladder, renal pelvis, basal-cell skin, and prostate cancer, and the non-invasive tumour types were rectal, bladder, and meningeal tumour. Altogether, the nine patients had 19 tumour diagnoses.

To examine the cancer risks for the heterozygous carriers, we retrieved the results for additive risk association analyses from FinnGen for the available malignant tumour types, which have been diagnosed in the patients with biallelic *NTHL1* variants in the FinnGen data or reported previously^6–11,13^. No increased risk of cancer was suggested for the heterozygous carriers for other cancer types than BC (Table 3, Supplementary Table S4).

## Discussion

We have performed a WES study of BC patients and a gnomAD database analysis of pLoF variants, with the aim of identifying novel BC risk variants. Furthermore, a recent exome-sequencing study of Finnish patients identified *SERPINA3* as a novel candidate gene for moderate-risk BC predisposition^19^. We assessed the cancer risk associated with the candidate variants by evaluating them in series of BC patients and controls from the Helsinki and Tampere regions and from the FinnGen project.

Even though we did not detect a significant association between *NTHL1* c.244C>T p.(Gln82Ter) and BC risk in our patient series, a much larger genotype dataset from FinnGen showed a high increased risk of BC for homozygous (OR = 44.7, P = 6.7 × 10^–5^) and a low increased risk for heterozygous women (OR = 1.39, P = 7.8 × 10^–5^). Different cancer studies have reported a high frequency of BC (55%) among women with biallelic pathogenic *NTHL1* variants, as reviewed by Beck et al.^6–13^. The association of *NTHL1* variants with BC predisposition has previously been evaluated in a large international case–control study; however, just one biallelic patient was identified and the BC risk remained unclear also for the heterozygous carriers^25^. In that study, the carrier frequencies and associated BC risk for the c.244C>T variant varied between patient series, but the results for other, rarer heterozygous pLoF and pathogenic missense variants suggested a low increased risk of BC^25^. The c.244C>T variant (previously reported as c.268C>T p.(Gln90Ter)) is the most frequent LoF variant identified in the patients with *NTHL1* tumour syndrome as well as in the *NTHL1* gene in the gnomAD database^13,26^. The variant is enriched in the uniform Finnish population—it was found with a minor allele frequency (MAF) of 0.0044 in the controls from the FinnGen study—which facilitates the detection of increased risk.

Biallelic pathogenic variants in the *NTHL1* gene cause a high-penetrance multi-tumour syndrome, which is especially manifested with colorectal, breast, endometrial, urothelial, and basal-cell skin cancer, as well as meningeal tumours^6–13^. Of the previously reported homozygous and compound heterozygous individuals, 49% had CRC, and of the individuals who had undergone a colonoscopy, even 93% had colonic adenomas^13^. The FinnGen results support the previous findings on high risk of CRC for the individuals with biallelic variants^6,9–11,13^. The present study also indicates a high recessive risk of BC; furthermore, high risks of basal-cell skin carcinoma and urinary tract cancer are suggested. Combining the FinnGen and the Helsinki patient series, 11 out of the identified 12 homozygous individuals had a total of 24 tumour diagnoses, further supporting high-penetrance cancer risk. Other cancer types, which have been reported in more than one biallelic case, include hematologic malignancies, squamous cell carcinomas of the head and neck, thyroid, pancreatic, and prostate cancer, and melanoma^6,7,9–11,13^.

Monoallelic *NTHL1* variants are unlikely to cause a substantially increased risk of cancer if any^8,12,25,27^. In the current study, we examined the risks for the heterozygous carriers to malignant tumours, which have been detected in the patients with biallelic *NTHL1* variants^6–13^. We observed no increased risk of any other cancer type than BC; however, for some tumour types, the case groups were small. In addition to BC, the risk associated with monoallelic *NTHL1* variants has previously been investigated in CRC, polyposis, and in a pan-cancer patient population^8,12,27^. In line with our results, no increased risk of other cancer types was detected.

The premature stop codon caused by the *NTHL1* c.244C>T variant has been reported to activate the nonsense-mediated mRNA decay surveillance mechanism^6^, resulting in loss of the *NTHL1* gene product in homozygous individuals. Consistently, reduced NTHL1 protein expression has been observed in heterozygous carriers^25^. The NTHL1 protein is a bifunctional DNA glycosylase, which catalyses the initial step of base excision-repair pathway to remove oxidative DNA damage^28–30^. NTHL1 has glycosylase activity on damaged bases, with a preference for oxidized pyrimidines as the substrate, and apurinic/apyrimidinic lyase activity on the DNA phosphate backbone^28,29^. Disruption of the NTHL1 function may lead to mispairing of damaged bases in replication and accumulation of sequence-specific mutations^30^. Biallelic LoF variants in the *NTHL1* gene have been shown to drive a mutational process causing the COSMIC signature SBS30, which is characterized by somatic C>T transitions at non-CpG sites over different tumour types, including BC^6,9,12,25,31,32^. Although there is some contradiction, the mutational signature 30, somatic loss of a second allele, or promoter methylation have typically not been observed in heterozygous *NTHL1* variant carriers^12,25,27,32,33^—in these individuals, the possible increased risk of cancer has been suggested to be caused by haploinsufficiency^25^.

The current study is a comprehensive cancer risk analysis for *NTHL1* in an extensive case–control material. Previous studies have been unable to estimate the associated risks for the biallelic individuals in a case–control setting. In the FinnGen data, the prevalence of individuals homozygous for the *NTHL1* c.244C>T variant was 1 in every 41,200. This is higher than the estimate of 1 in 114,770 Europeans^30^. Still, due to the rarity of homozygous individuals, the observed effect sizes for the increased recessive risk associated with the c.244C>T variant, here, are uncertain and the CIs are wide, and the *NTHL1* gene warrants further evaluation for more precise risk estimates for different cancer types. Nevertheless, because of the high cancer risk, we suggest that *NTHL1* should be included in cancer gene panels in clinical diagnostics, at least for the most common tumour types reported in the patients with pathogenic biallelic *NTHL1* variants. Additionally, the susceptibility to multiple tumour types should be considered in surveillance and cancer-prevention strategies for the individuals with biallelic variants, and clinical practice guidelines should be developed for the *NTHL1* gene.

*FANCG* c.1182_1192delinsC p.(Glu395TrpfsTer5) was rare in our patient series, and it was not included in the FinnGen dataset; hence, we were unable to statistically assess any BC risk associated with it. *FANCG* is an established FA risk gene, with p.(Glu395fs) among the first described causative *FANCG* mutations for the syndrome^20,21^. Monoallelic variants in several FA genes are known to predispose to BC^3^. Two other *FANCG* pLoF variants, c.832insG p.(Ala278GlyfsTer11) and c.1076+1G>A, identified in the BC patients in the FinnGen study, have been discovered also in Finnish FA patients^34^. No association with increased risk of BC was detected for these two variants in the FinnGen data; however, both variants were very rare in the study subjects. We did not find heterozygous *ERCC6L2* variants associated with BC risk. The additive ORs were inconsistent between the different *ERCC6L2* variants in the FinnGen data, which may have been influenced by the rarity of the variants. Biallelic LoF variants in the *ERCC6L2* gene, including homozygous c.1424del p.(Ile475ThrfsTer36) (previously known as c.1457del), have been described in patients with inherited bone marrow failure and acute myeloid leukaemia^22,23^. Additionally, a BC patient with biallelic variants has been reported^23^. The homozygous c.1424del variant was detected among the BC patients also in the current study, and the contribution of *ERCC6L2* to BC remains unclear.

We identified *MAD1L1* c.1947C>G p.(Tyr649Ter) and *USP45* c.2190C>A p.(Tyr730Ter) in about four- to fivefold higher frequency in the unselected patient group compared with the controls from the Helsinki and Tampere regions. A recent copy number variant analysis reported a twofold increased frequency of *MAD1L1* gene deletions among patients in a large BC dataset^35^; additionally, p.(Tyr649Ter) has been suggested to have a dominant-negative effect on the MAD1L1 protein function and impair the mitotic spindle-assembly checkpoint^36^. Other studies have connected *USP45* to hypersensitivity to mitomycin C -induced interstrand crosslinks and as a candidate gene to multiple myeloma^37,38^. Our results did not remain significant after adjusting the P value threshold for multiple comparisons and no association with BC risk was detected for the *MAD1L1* and *USP45* genes in the FinnGen data. We found the *SERPINA3* c.918-1G>C variant with a similar frequency in the BC patients and in the controls both in the Helsinki and Tampere BC series and in the FinnGen data; therefore, in the current study, no association with increased BC risk was detected.

In conclusion, our results indicate that biallelic LoF variants in the *NTHL1* gene cause a high risk of multiple cancer types, including BC. We also suggest *NTHL1* as a low-risk gene for BC predisposition in heterozygous women. However, further studies are required to estimate the effect sizes for the increased risk of different cancer types more precisely. Finally, we propose that *NTHL1* should be included in cancer gene panels in clinical diagnostics and clinical practice guidelines should be developed for cancer screening strategies for individuals with pathogenic biallelic *NTHL1* variants.

## Materials and methods

### Whole-exome sequencing and variant calling

We included 69 BC patients from 44 families in the WES. Of the families, 39 had at least three patients with BC or OC among first- and second-degree relatives and 4 had two affected first-degree relatives. Furthermore, 10 of the families included male BC patients, 19 families had uterine cancer cases, and 8 families were suspected of Li-Fraumeni-like syndrome. None of the exome-sequenced patients had a pathogenic *BRCA1/2* or *TP53* variant. The index patients and their family members were collected among the Helsinki BC series as described below. The WES was carried out using genomic DNA extracted from peripheral blood samples.

The sequencing and variant calling was performed at the McGill University and Génome Québec Innovation Centre, Montreal, Canada. Exome libraries were created with Roche Nimblegen SeqCap EZ Exome + UTR capture kit for 39 of the samples and Roche Nimblegen SeqCap EZ Exome v3 kit for 30 of the samples. Sequencing of the libraries was performed with Illumina HiSeq 2000 platform with 100 bp paired-end reads. The read quality trimming of FASTQ files was executed with FASTX-toolkit (http://hannonlab.cshl.edu/fastx_toolkit/). The reads were aligned to the human reference genome GRCh37/hg19 with Burrows-Wheeler Aligner^39^. Insertion and deletion variants (indels) were realigned and duplicates were marked with Picard (https://broadinstitute.github.io/picard/). The metrics were computed with Integrative Genomics Viewer^40^, and the variant calling was performed with SAMtools and BCFtools^41,42^.

### Variant selection from the whole-exome sequencing data

The candidate variants were selected for genotyping based on MAF, pathogenicity of the variant, and relevant gene function. We annotated the variants with Annovar^43^ and retrieved gene ontology (GO) terms from the AmiGO2 website by the Gene Ontology Consortium^44,45^. We excluded variants with a raw read depth of < 30 and a phred-scaled quality probability of < 10. Common variants with a MAF of > 0.03 were excluded using the Exome Aggregation Consortium (in any population) and the 1000Genomes variant databases^46,47^. This selection stage yielded 22,531 variants, which were predicted to alter the protein sequence. We included pLoF variants, defined as stop-gain, frameshift, and essential splice site variants, involved in DNA repair (GO:0006281), cell cycle (GO:0007049), or apoptotic pathways (GO:0006915), totalling in 178 variants in 160 genes. PLoF variants outside of these pathways were considered based on relevance in tumorigenesis. Missense variants involved in DNA repair or cell cycle pathways were considered if predicted to be pathogenic by CADD^48^ (phred ≥ 20) and by the majority of the other pathogenicity prediction tools included in the LJB (dbNSFP) database in Annovar^43^ (201 variants in 174 genes). Finally, we focused on plausible candidate genes based on gene function, queried from the UniProt and the NCBI Gene databases^49,50^, and selected 28 variants in well-supported transcripts^51^ for genotyping, including 21 pLoFs and seven missenses (Supplementary Table S1). All selected variants had a raw read depth of ≥ 600 and a phred-scaled quality probability of ≥ 150 in the WES data. We further confirmed the indel variants with Sanger sequencing. The variant descriptions were confirmed with Mutalyzer 3 and comply with the current HGVS nomenclature^52,53^.

### Variant selection from the gnomAD database

We downloaded the exomes data of approximately 11,000 Finns from the gnomAD database, release 2.0.1, for about 520 DNA repair genes (GO:0006281, release 2017-07-01)^26,44,45^. We selected only high-confidence pLoF variants with a MAF of 0.0001–0.03 in the Finnish population; furthermore, we excluded the variants with a MAF of > 0.03 in any other population. We excluded the variants in the validated BC risk genes and in the candidate risk genes previously published from the Helsinki BC series^3,54,55^. This selection stage yielded 124 pLoF variants in 92 genes in well-supported transcripts (transcript support level 1 and 2), annotated with transcript flags from the Ensembl database through BioMart^51,56^. We prioritized the candidate variants based on gene function^49,50^, similarly as for variants chosen from the WES data, and selected twelve pLoF variants in ten candidate genes for genotyping (Supplementary Table S1).

### Patient and control series

The case–control series included a total of 4101 BC patients and 3985 population controls from the Helsinki and Tampere regions. All study subjects from Helsinki were women, whereas the Tampere control group also included men. The genomic DNA used in genotyping had been extracted from peripheral blood samples.

### Breast cancer patients

The unselected Helsinki BC series consisted of 1726 patients who had been diagnosed with their first primary invasive BC. The patients were recruited consecutively in the Helsinki University Hospital at the Department of Oncology in 1997–1998 and 2000 (n = 847) and at the Department of Surgery in 2001–2004 (n = 879)^18,57,58^ without any selection criteria for family history of BC or age of diagnosis. The familial Helsinki BC series was combined from 380 index patients with a family history of BC or OC from the unselected series and from 756 additional index patients who were recruited at the Department of Oncology and at the Department of Clinical Genetics until 2015^58–60^. Of these 1136 familial patients, 606 had a family history of at least three BC or OC patients among first- or second-degree relatives (including the proband) and 530 had one affected first-degree relative. The familial patients had been tested at least for *BRCA1/2* founder mutations in Finland and the carriers had been excluded from the series. The cancer diagnoses of the patients and their family members were confirmed from hospital records and/or the Finnish Cancer Registry. Altogether, the Helsinki BC series included a total of 2482 patients.

Additional unselected BC patients from the Helsinki region, the BrePainGen series, had been collected in the Helsinki University Hospital at the Breast Surgery Unit in 2006–2010^61^. The series consisted of 950 patients with invasive breast tumour, which had been unilateral and non-metastasised at the time of recruitment; however, no selection for family history of the disease or age of diagnosis had been performed. Of the patients, 161 had at least one first- or second-degree relative diagnosed with BC or OC and were classified as familial.

The unselected Tampere BC series consisted of 669 patients who had been recruited at the Tampere University Hospital consecutively in 1997–1999 and additionally in 1996–2004^18,58^. All patients had been newly diagnosed with invasive BC. Altogether 234 patients had at least one first- or second-degree relative diagnosed with BC or OC and were defined familial.

### Population controls

The geographically matched population controls from the Helsinki region consisted of 1273 anonymous blood donors, collected in 2002–2003, and 1896 additional controls with no cancer diagnosis from the Helsinki Biobank. The population controls from the Tampere region consisted of 816 blood donors.

### Variant genotyping

Twenty-one variants selected from the WES data were genotyped in 3143 BC patients and 2089 controls from the Helsinki and Tampere BC series with the Sequenom MassARRAY. Seven indel variants from the WES data were genotyped outside of the array for technical reasons. Changes of ≤ 6 base pairs were genotyped with TaqMan real-time PCR and larger indels with 3% agarose gel electrophoresis in 2482 BC patients and 1273 controls from Helsinki. Positive control samples were included in all analyses and the carriers detected with 3% agarose gel electrophoresis were confirmed with Sanger sequencing. Twelve variants selected from the gnomAD data were genotyped in 2482 BC patients and 1273 controls from Helsinki with the Sequenom MassARRAY.

The genotyping of four variants, which had been analysed in the Helsinki BC series, was continued to the 669 BC patients and 816 controls of the Tampere BC series. We genotyped *ERCC6L2* c.1424del and *USP45* c.2190C>A with TaqMan real-time PCR, *USP45* c.1008del with Sanger sequencing, and *FANCG* c.1182_1192delinsC with 3% agarose gel electrophoresis. The genotyping of *MAD1L1* c.1947C>G was further continued to additional 950 BC patients from the BrePainGen series and 1896 controls from the Helsinki Biobank with TaqMan real-time PCR. *SERPINA3* c.918-1G>C, selected for genotyping outside of the WES or the gnomAD variant data, was genotyped in all 4101 BC patients and 3985 controls with TaqMan real-time PCR. We confirmed the detected carriers for the *ERCC6L2* c.1424del, *FANCG* c.1182_1192delinsC, *MAD1L1* c.1947C>G, *NTHL1* c.244C>T, *SERPINA3* c.918-1G>C, and *USP45* c.2190C>A and c.1008del variants with Sanger sequencing. Further details on genotyping are given in the Supplementary Information Methods.

### Statistical analyses

We performed the statistical analyses using the R environment for statistical computing (version 4.2.2)^62^. We used region-adjusted logistic regression for the combined analyses including patients from Helsinki and Tampere BC series and Fisher’s exact test for the Helsinki BC series, with two-sided P values. After the Bonferroni correction for multiple comparisons, P values < 0.0012 were considered statistically significant.

### FinnGen data

To further evaluate the candidate genes, we obtained the data for cancer risk association analyses for a total of 412,181 individuals (230,310 women and 181,871 men) from the FinnGen research project (https://www.finngen.fi/en), which produces genotype data from samples of Finnish biobank participants and combines it with longitudinal data from Finnish health registries^24^. The biobank sample and data accession numbers for the FinnGen data release 10 are presented in the Supplementary Information Materials.

We retrieved the results for BC risk association analyses for all 38 candidate genes with the endpoint C3_BREAST_EXALLC, which included 18,786 female BC patients and 182,927 female controls with no cancer diagnosis. We annotated the variants with Annovar^43^; from these results, we included pLoF, missense, and in-frame indel variants with a MAF of ≤ 0.03 in the controls. Additionally, we retrieved the data for risk association analyses for all available tumour types, which had been detected in cancer patients with biallelic pathogenic variants in the *NTHL1* gene in the FinnGen study and in previous reports^6–13^. We excluded the endpoints for benign and in situ tumours (ICD-10 D-coded tumours), as the registry entries may be incomplete for them, except for the endpoint C3_BREAST_EXALLC, which included both malignant and in situ tumours (ICD-O-3 behaviour codes 3 and 2). We used the analyses in which the controls with any cancer diagnosis had been excluded. All included cancer endpoints are given in the Supplementary Table S5 and the endpoint definitions are available at https://risteys.finregistry.fi.

The cancer risk associated with heterozygous variants was detected with the additive model in the FinnGen data; homozygous and compound heterozygotes had been excluded from the analyses as described in^15^. The recessive model compared homozygous individuals against heterozygotes and noncarriers^15^. Of the additive analyses, we included only variants which had been genotyped on array, whereas the recessive analyses for *NTHL1* c.244C>T and *ERCC6L2* c.1424del included also imputed genotypes. The imputation quality scores were 0.9974 for *NTHL1* c.244C>T and 0.9951 for *ERCC6L2* c.1424del. The association analyses in the FinnGen data had been performed with the REGENIE software (version 2.2.4)^63^. The genotyping and production of the FinnGen dataset has been described in^24^ and at https://finngen.gitbook.io/documentation.

### Ethics declarations

The study was conducted in accordance with the Declaration of Helsinki and with approval by the Ethics Committee of the Helsinki University Hospital (Dnro207/E9/07 and HUS71597/2016). The Tampere study protocol was approved by the Ethics Committee of the Pirkanmaa Hospital District (97247) and the BrePainGen study protocol by the Coordinating Ethics Committee (136/E0/2006) and the Ethics Committee of the Department of Surgery (Dnro 148/E6/05) of the Hospital District of Helsinki and Uusimaa. The ethics statement for FinnGen is given in the Supplementary Information Materials. Informed consent was obtained from all patients.

### Supplementary Information

Supplementary Information 1.

Supplementary Table S3.

Supplementary Table S6.

## Data Availability

For the Helsinki and Tampere BC series, the data that support the findings of our study are available on reasonable request from the corresponding author. The data are not publicly available due to privacy or ethical restrictions. Instructions on accessing the FinnGen data are available at https://www.finngen.fi/en/access_results.
