# Supplementary material for: NTHL1 is a recessive cancer susceptibility gene
Source: Sci Rep. 2023 Nov 30;13:21127. doi: 10.1038/s41598-023-47441-w (PMC10689455; doi:10.1038/s41598-023-47441-w)
Supplement: Supplementary file 1 — Supplementary Information 1. [file 41598_2023_47441_MOESM1_ESM.pdf]

NTHL1 IS A RECESSIVE CANCER SUSCEPTIBILITY GENE

Anna K. Nurmi, Liisa M. Pelttari, Johanna I. Kiiski, Sofia Khan, Mika Nurmikolu, Maija Suvanto, Niina Aho, Tiina Tasmuth, Eija Kalso, Johanna Schleutker, Anne Kallioniemi, Päivi Heikkilä, FinnGen, Kristiina Aittomäki, Carl Blomqvist, Heli Nevanlinna

SUPPLEMENTARY INFORMATION MATERIALS

Supplementary Table S1: Candidate variants

Supplementary Table S2: Variant frequencies in the Helsinki and Tampere breast cancer series

Supplementary Table S4: Risk association analyses of additional cancer types from FinnGen for heterozygous *NTHL1* c.244C>T carriers

Supplementary Table S5: Cancer endpoints from FinnGen

Supplementary Information Methods: Genotyping

Biobank sample and data accession numbers for FinnGen

Ethics statement for FinnGen

Supplementary Table S1: Candidate variants

Source	Location	Gene	Variant (HGVS name)	Transcript (RefSeq)	Transcript (Ensembl)	Rs number	All MAF	FIN MAF	GO term
gnomAD	Chr1:35745544	*CLSPN*	c.2873C>A p.(Ser958Ter)	NM_022111.4	ENST00000318121.8	rs778356330	2.00×10^{-4}	2.60×10^{-3}	DNA repair, cell cycle
WES	Chr1:46274607	*RAD54L*	c.1759C>T p.(Arg587Trp)	NM_003579.4	ENST00000371975.9	rs150315374	3.70×10^{-3}	2.92×10^{-2}	DNA repair, cell cycle
WES	Chr1:182586014	*RNASEL*	c.793G>T p.(Glu265Ter)	NM_021133.4	ENST00000367559.7	rs74315364	3.60×10^{-3}	8.20×10^{-3}	Other
WES	Chr2:28310080	*BABAM2*	c.1089-2A>C	NM_199193.3	ENST00000379632.6	rs150302537	2.80×10^{-3}	2.00×10^{-3}	DNA repair, cell cycle, apoptotic process
WES	Chr2:189795808	*PMS1*	c.172G>A p.(Gly58Arg)	NM_000534.5	ENST00000441310.7	rs772427166	1.00×10^{-4}	6.00×10^{-4}	DNA repair
WES	Chr3:8948567	*RAD18*	c.137G>A p.(Cys46Tyr)	NM_020165.4	ENST00000264926.7	rs746085217	4.08×10^{-5}	2.00×10^{-4}	DNA repair
WES	Chr3:121488663	*POLQ*	c.4262_4268del p.(Ile1421ArgfsTer8)	NM_199420.4	ENST00000264233.6	rs546221341	5.50×10^{-3}	6.00×10^{-3}	DNA repair
WES	Chr3:149071333	*HLTF*	c.813C>G p.(Tyr271Ter)	NM_003071.4	ENST00000310053.10	rs140317783	7.00×10^{-4}	6.70×10^{-3}	DNA repair
WES	Chr3:151294206	*GPR87*	c.1040C>A p.(Ser347Ter)	NM_023915.4	ENST00000260843.5	rs142901780	9.00×10^{-4}	2.10×10^{-3}	Other
gnomAD	Chr4:1348146	*UVSSA*	c.55C>T p.(Arg19Ter)	NM_020894.4	ENST00000389851.10	rs199574083	4.87×10^{-5}	4.00×10^{-4}	DNA repair
gnomAD	Chr4:184691582	*PRIMPOL*	c.1378+1G>C	NM_152683.4	ENST00000314970.11	rs144707273	8.14×10^{-5}	9.00×10^{-4}	DNA repair
gnomAD	Chr6:99437370	*USP45*	c.2190C>A p.(Tyr730Ter)	NM_001346022.3	ENST00000500704.7	rs118066385	1.20×10^{-3}	1.30×10^{-3}	DNA repair
gnomAD	Chr6:99468544	*USP45*	c.1008del p.(Val337SerfsTer9)	NM_001346022.3	ENST00000500704.7	rs554927779	1.90×10^{-3}	7.00×10^{-4}	DNA repair
WES	Chr7:1898251	*MAD1L1*	c.1947C>G p.(Tyr649Ter)	NM_001013836.2	ENST00000265854.12	rs121908981	3.66×10^{-5}	2.00×10^{-4}	Cell cycle
WES	Chr8:17755721	*MTUS1*	c.87C>G p.(Tyr29Ter)	NM_001363059.2	ENST00000693296.1	rs181601359	1.60×10^{-3}	6.10×10^{-3}	Other
WES	Chr8:106726258	*OXR1*	c.15G>A p.(Trp5Ter)	NM_001198534.1	ENST00000297447.10	rs145739822	3.90×10^{-3}	6.50×10^{-3}	Apoptotic process
WES	Chr8:144515324	*RECQL4*	c.1390+2del	NM_004260.4	ENST00000617875.6	rs386833843	4.00×10^{-4}	4.00×10^{-3}	DNA repair
WES	Chr9:35075706	*FANCG*	c.1182_1192delinsC p.(Glu395TrpfsTer5)	NM_004629.2	ENST00000378643.8	rs397507559	7.17×10^{-5}	1.73×10^{-4}	DNA repair
gnomAD	Chr9:95923270	*ERCC6L2*	c.1424del p.(Ile475ThrfsTer36)	NM_020207.7	ENST00000653738.2	rs768081343	5.00×10^{-4}	5.20×10^{-3}	DNA repair
gnomAD	Chr10:49461473	*ERCC6*	c.3862C>T p.(Arg1288Ter)	NM_000124.4	ENST00000355832.10	rs185142838	1.00×10^{-4}	1.30×10^{-3}	DNA repair, apoptotic process
WES	Chr10:87862148	*KLLN*	c.339_340del p.(Ala115SerfsTer58)	NM_001126049.2	ENST00000445946.5	rs749052307	1.40×10^{-3}	5.10×10^{-3}	Cell cycle, apoptotic process
gnomAD	Chr11:47232931	*DDB2*	c.574C>T p.(Arg192Ter)	NM_000107.3	ENST00000256996.9	rs199822504	6.09×10^{-5}	6.00×10^{-4}	DNA repair
WES	Chr11:104944848	*CASP4*	c.1039C>T p.(Gln347Ter)	NM_001225.4	ENST00000444739.7	rs148710034	7.00×10^{-4}	5.70×10^{-3}	Apoptotic process
gnomAD	Chr11:117381699	*CEP164*	c.1410-2A>G	NM_014956.5	ENST00000278935.8	rs200074826	7.00×10^{-4}	6.70×10^{-3}	DNA repair, cell cycle
WES	Chr12:48134277	*PFKM*	c.638+1G>T	NM_000289.6	ENST00000359794.11	rs766350964	8.12×10^{-6}	8.97×10^{-5}	Other
WES	Chr13:72745001	*BORA*	c.532G>A p.(Glu178Lys)	NM_024808.5	ENST00000390667.11	rs182782800	1.60×10^{-3}	1.20×10^{-3}	Cell cycle
WES	Chr14:24172747	*REC8*	c.91C>T p.(Arg31Cys)	NM_001048205.2	ENST00000611366.5	rs34075659	3.00×10^{-3}	5.00×10^{-3}	DNA repair, cell cycle
gnomAD	Chr14:75039918	*MLH3*	c.3563C>G p.(Ser1188Ter)	NM_001040108.2	ENST00000355774.7	rs193219754	3.00×10^{-4}	2.50×10^{-3}	DNA repair, cell cycle
gnomAD	Chr14:75049209	*MLH3*	c.447del p.(Tyr149Ter)	NM_001040108.2	ENST00000355774.7	rs760973900	5.69×10^{-5}	6.00×10^{-4}	DNA repair, cell cycle

Supplementary Table S1 continues

Source	Location	Gene	Variant (HGVS name)	Transcript (RefSeq)	Transcript (Ensembl)	Rs number	All MAF	FIN MAF	GO term
Additional	Chr14:94622340	*SERPINA3*	c.918-1G>C	NM_001085.5	ENST00000393078.5	rs199710314	3.29×10^{-4}	3.70×10^{-3}	Other
WES	Chr15:30905592	*FAN1*	c.929C>G p.(Ser310Ter)	NM_014967.5	ENST00000362065.9	rs201220536	1.00×10^{-4}	6.00×10^{-4}	DNA repair
WES	Chr15:89301393	*FANCI*	c.2957_2969del p.(Val986AlafsTer39)	NM_001113378.2	ENST00000310775.12	rs762390984	3.00×10^{-4}	2.90×10^{-3}	DNA repair, cell cycle
WES	Chr16:79695	*MPG*	c.295G>A p.(Gly99Arg)	NM_001015052.3	ENST00000356432.8	rs776034664	5.00×10^{-4}	5.00×10^{-3}	DNA repair
WES	Chr16:2046238	*NTHL1*	c.244C>T p.(Gln82Ter)	NM_002528.7	ENST00000651570.2	rs150766139	1.40×10^{-3}	3.80×10^{-3}	DNA repair
gnomAD	Chr16:27235190	*NSMCE1*	c.246del p.(Ile83PhefsTer5)	NM_145080.4	ENST00000361439.9	rs748904364	1.00×10^{-4}	1.20×10^{-3}	DNA repair
WES	Chr17:7436176	*TMEM102*	c.215-17_218del	NM_178518.3	ENST00000323206.2	rs537361079	6.49×10^{-3}	5.82×10^{-3}	Apoptotic process
WES	Chr17:8047006	*ALOX15B*	c.1387C>T p.(Arg463Ter)	NM_001141.3	ENST00000380183.9	rs139880289	5.00×10^{-4}	9.00×10^{-4}	Apoptotic process
WES	Chr17:76385966	*SPHK1*	c.35_36del p.(Phe12TrpfsTer46)	NM_021972.4	ENST00000590959.5	rs746915055	1.38×10^{-3}	3.96×10^{-4}	Other
WES	Chr17:76387027	*SPHK1*	c.596del p.(Arg199LeufsTer17)	NM_001142601.2	ENST00000592299.6	rs549579958	2.20×10^{-3}	1.30×10^{-3}	Other
WES	Chr19:4328758	*STAP2*	c.507C>G p.(Tyr169Ter)	NM_001013841.2	ENST00000594605.6	rs79657645	9.10×10^{-3}	8.50×10^{-3}	Other
WES	Chr20:47183374	*EYA2*	c.1519G>C p.(Glu507Gln)	NM_005244.5	ENST00000327619.10	rs137929907	4.00×10^{-4}	5.00×10^{-4}	DNA repair, apoptotic process

The genomic locations are indicated according to the human genome reference build GRCh38/hg38. The minor allele frequencies (MAFs) for all populations and the Finns were retrieved from the Genome Aggregation Database (gnomAD).

WES, whole-exome sequencing; HGVS, Human Genome Variation Society; GO, the Gene Ontology

Supplementary Table S2: Variant frequencies in the Helsinki and Tampere breast cancer series

Variant	Series	Total	Carriers	%	OR	95% CI	P value
ALOX15B	Controls	2086	7	0.3			
c.1387C>T p.(Arg463Ter)	All BC	3128	13	0.4	1.39	0.56–3.77	0.49
rs139880289	*Familial BC*	1360	5	0.4	1.25	0.36–4.11	0.71
	Unselected BC	2378	9	0.4	1.23	0.45–3.48	0.68
	ER-positive BC	2375	8	0.3	1.10	0.39–3.21	0.86
	ER-negative BC	574	4	0.7	2.51	0.64–8.59	0.15
BABAM2	Controls	2077	9	0.4			
c.1089-2A>C	All BC	3119	18	0.6	1.27	0.58–3.01	0.56
rs150302537	*Familial BC*	1357	10	0.7	1.68	0.66–4.37	0.27
	Unselected BC	2369	12	0.5	1.15	0.48–2.83	0.76
	ER-positive BC	2369	14	0.6	1.24	0.54–3.01	0.62
	ER-negative BC	572	4	0.7	1.68	0.45–5.30	0.40
BORA	Controls	2083	4	0.2			
c.532G>A p.(Glu178Lys)	All BC	3124	9	0.3	1.16	0.38–4.30	0.80
rs182782800	*Familial BC*	1357	5	0.4	1.41	0.37–5.72	0.61
	Unselected BC	2374	5	0.2	0.93	0.25–3.76	0.91
	ER-positive BC	2372	8	0.3	1.36	0.43–5.12	0.61
	ER-negative BC	574	1	0.2	0.71	0.04–4.78	0.76
CASP4	Controls	2084	23	1.1			
c.1039C>T p.(Gln347Ter)	All BC	3123	33	1.1	0.94	0.55–1.64	0.83
rs148710034	*Familial BC*	1359	15	1.1	1.01	0.51–1.97	0.97
	Unselected BC	2372	22	0.9	0.84	0.46–1.52	0.56
	ER-positive BC	2372	25	1.1	0.95	0.53–1.70	0.86
	ER-negative BC	572	8	1.4	1.28	0.53–2.80	0.55
CEP164	Controls	1270	11	0.9			
c.1410-2A>G [1]	All BC	2474	18	0.7	0.84	0.37–1.97	0.69
rs200074826	*Familial BC*	1135	8	0.7	0.81	0.28–2.23	0.82
	Unselected BC	1719	11	0.6	0.74	0.29–1.88	0.52
	ER-positive BC	1874	14	0.7	0.86	0.36–2.10	0.84
	ER-negative BC	449	3	0.7	0.77	0.14–2.93	1.00
CLSPN	Controls	1247	1	0.1			
c.2873C>A p.(Ser958Ter) [1]	All BC	2427	4	0.2	2.06	0.20–101	0.67
rs778356330	*Familial BC*	1115	2	0.2	2.24	0.12–132	0.61
	Unselected BC	1687	3	0.2	2.22	0.18–117	0.64
	ER-positive BC	1845	3	0.2	2.03	0.16–107	0.65
	ER-negative BC	436	1	0.2	2.86	0.04–225	0.45
DDB2	Controls	1272	0	0.0			
c.574C>T p.(Arg192Ter) [1]	All BC	2474	1	0.04	NA	NA	1.00
rs199822504	*Familial BC*	1135	0	0.0	NA	NA	NA
	Unselected BC	1719	1	0.1	NA	NA	1.00
	ER-positive BC	1874	1	0.1	NA	NA	1.00
	ER-negative BC	449	0	0.0	NA	NA	NA
ERCC6	Controls	1245	4	0.3			
c.3862C>T p.(Arg1288Ter) [1]	All BC	2409	6	0.2	0.77	0.18–3.74	0.74
rs185142838	*Familial BC*	1112	2	0.2	0.56	0.05–3.91	0.69
	Unselected BC	1663	5	0.3	0.94	0.20–4.73	1.00
	ER-positive BC	1820	4	0.2	0.68	0.13–3.68	0.72
	ER-negative BC	439	2	0.5	1.42	0.13–9.94	0.65

Supplementary Table S2 continues

Variant	Series	Total	Carriers	%	OR	95% CI	P value
EYA2	Controls	2057	6	0.3			
c.1519G>C p.(Glu507Gln)	All BC	3001	3	0.1	0.31	0.07–1.21	0.10
rs137929907	*Familial BC*	1253	3	0.2	0.74	0.15–2.91	0.68
	Unselected BC	2347	0	0.0	NA	NA	0.95
	ER-positive BC	2297	3	0.1	0.41	0.09–1.58	0.21
	ER-negative BC	538	0	0.0	NA	NA	0.97
FAN1	Controls	2083	2	0.1			
c.929C>G p.(Ser310Ter)	All BC	3128	2	0.1	0.79	0.09–6.87	0.82
rs201220536	*Familial BC*	1361	0	0.0	NA	NA	0.96
	Unselected BC	2377	2	0.1	0.95	0.11–8.04	0.96
	ER-positive BC	2375	0	0.0	NA	NA	0.95
	ER-negative BC	574	2	0.3	4.32	0.50–37.4	0.15
FANCI	Controls	1273	3	0.2			
c.2957_2969del [1]	All BC	2479	10	0.4	1.71	0.44–9.71	0.56
p.(Val986AlafsTer39)	*Familial BC*	1133	5	0.4	1.88	0.36–12.1	0.49
rs762390984	*Unselected BC*	1725	8	0.5	1.97	0.47–11.6	0.37
	ER-positive BC	1878	9	0.5	2.04	0.51–11.7	0.38
	ER-negative BC	452	1	0.2	0.94	0.02–11.7	1.00
GPR87	Controls	2084	7	0.3			
c.1040C>A p.(Ser347Ter)	All BC	3127	10	0.3	1.01	0.38–2.84	0.98
rs142901780	*Familial BC*	1360	6	0.4	1.47	0.46–4.62	0.51
	Unselected BC	2376	8	0.3	1.04	0.37–2.99	0.94
	ER-positive BC	2374	7	0.3	0.98	0.33–2.93	0.97
	ER-negative BC	574	2	0.3	1.12	0.16–4.77	0.89
HLTF	Controls	2086	27	1.3			
c.813C>G p.(Tyr271Ter)	All BC	3126	57	1.8	1.37	0.87–2.23	0.18
rs140317783	*Familial BC*	1360	25	1.8	1.34	0.76–2.36	0.31
	Unselected BC	2376	45	1.9	1.44	0.89–2.36	0.14
	ER-positive BC	2373	47	2.0	1.47	0.91–2.42	0.12
	ER-negative BC	574	8	1.4	1.00	0.42–2.14	1.00
KLLN	Controls	1272	15	1.2			
c.339_340del [1]	All BC	2390	38	1.6	1.35	0.72–2.66	0.38
p.(Ala115SerfsTer58)	*Familial BC*	1044	16	1.5	1.30	0.60–2.85	0.47
rs749052307	*Unselected BC*	1726	29	1.7	1.43	0.74–2.89	0.29
	ER-positive BC	1820	29	1.6	1.36	0.70–2.73	0.36
	ER-negative BC	431	8	1.9	1.58	0.58–4.01	0.33
MLH3	Controls	1269	0	0.0			
c.447del p.(Tyr149Ter) [1]	All BC	2465	1	0.04	NA	NA	1.00
rs760973900	*Familial BC*	1128	0	0.0	NA	NA	NA
	Unselected BC	1717	1	0.1	NA	NA	1.00
	ER-positive BC	1867	1	0.1	NA	NA	1.00
	ER-negative BC	449	0	0.0	NA	NA	NA
MLH3	Controls	1264	6	0.5			
c.3563C>G p.(Ser1188Ter) [1]	All BC	2459	9	0.4	0.77	0.24–2.64	0.60
rs193219754	*Familial BC*	1131	3	0.3	0.56	0.09–2.62	0.51
	Unselected BC	1708	7	0.4	0.86	0.25–3.12	0.79
	ER-positive BC	1866	7	0.4	0.79	0.23–2.85	0.78
	ER-negative BC	443	2	0.5	0.95	0.09–5.34	1.00

Supplementary Table S2 continues

Variant	Series	Total	Carriers	%	OR	95% CI	P value
MPG	Controls	2061	14	0.7			
c.295G>A p.(Gly99Arg)	All BC	3001	28	0.9	1.30	0.69–2.58	0.43
rs776034664	*Familial BC*	1253	9	0.7	0.92	0.38–2.14	0.86
	Unselected BC	2348	21	0.9	1.27	0.65–2.58	0.49
	ER-positive BC	2297	18	0.8	1.08	0.53–2.24	0.83
	ER-negative BC	537	7	1.3	1.80	0.67–4.38	0.21
MTUS1	Controls	2085	24	1.2			
c.87C>G p.(Tyr29Ter)	All BC	3126	43	1.4	1.19	0.72–2.01	0.51
rs181601359	*Familial BC*	1358	25	1.8	1.55	0.87–2.78	0.14
	Unselected BC	2377	30	1.3	1.10	0.64–1.91	0.73
	ER-positive BC	2375	33	1.4	1.22	0.72–2.11	0.47
	ER-negative BC	573	6	1.0	0.89	0.33–2.08	0.81
NSMCE1	Controls	1270	4	0.3			
c.246del p.(Ile83PhefsTer5) [1]	All BC	2472	5	0.2	0.64	0.14–3.24	0.50
rs748904364	*Familial BC*	1135	3	0.3	0.84	0.12–4.97	1.00
	Unselected BC	1716	4	0.2	0.74	0.14–3.98	0.73
	ER-positive BC	1872	3	0.2	0.51	0.07–3.01	0.45
	ER-negative BC	449	1	0.2	0.71	0.01–7.17	1.00
OXR1	Controls	2075	22	1.1			
c.15G>A p.(Trp5Ter)	All BC	3112	32	1.0	1.05	0.60–1.85	0.87
rs145739822	*Familial BC*	1349	14	1.0	1.15	0.56–2.29	0.70
	Unselected BC	2364	24	1.0	1.01	0.56–1.82	0.98
	ER-positive BC	2364	23	1.0	1.02	0.56–1.87	0.95
	ER-negative BC	570	8	1.4	1.51	0.62–3.33	0.33
PFKM	Controls	2085	0	0.0			
c.638+1G>T	All BC	3120	4	0.1	NA	NA	0.94
rs766350964	*Familial BC*	1361	0	0.0	NA	NA	NA
	Unselected BC	2369	4	0.2	NA	NA	0.94
	ER-positive BC	2368	3	0.1	NA	NA	0.95
	ER-negative BC	573	1	0.2	NA	NA	0.96
PMS1	Controls	2065	1	0.05			
c.172G>A p.(Gly58Arg)	All BC	3003	5	0.2	3.18	0.50–61.7	0.30
rs772427166	*Familial BC*	1254	2	0.2	2.45	0.23–52.8	0.46
	Unselected BC	2349	3	0.1	2.56	0.32–52.1	0.42
	ER-positive BC	2298	4	0.2	3.42	0.49–68.0	0.28
	ER-negative BC	538	0	0.0	NA	NA	0.97
POLQ	Controls	1272	20	1.6			
c.4262_4268del [1]	All BC	2479	30	1.2	0.77	0.42–1.43	0.37
p.(Ile1421ArgfsTer8)	*Familial BC*	1133	12	1.1	0.67	0.30–1.45	0.29
rs546221341	*Unselected BC*	1725	19	1.1	0.70	0.35–1.38	0.33
	ER-positive BC	1878	21	1.1	0.71	0.36–1.38	0.34
	ER-negative BC	452	6	1.3	0.84	0.27–2.19	0.83
PRIMPOL	Controls	1268	3	0.2			
c.1378+1G>C [1]	All BC	2474	8	0.3	1.37	0.33–8.02	0.76
rs144707273	*Familial BC*	1136	1	0.1	0.37	0.01–4.64	0.63
	Unselected BC	1718	7	0.4	1.72	0.39–10.4	0.53
	ER-positive BC	1874	7	0.4	1.58	0.36–9.49	0.75
	ER-negative BC	449	0	0.0	NA	NA	0.57

Supplementary Table S2 continues

Variant	Series	Total	Carriers	%	OR	95% CI	P value
RAD18	Controls	2065	0	0.0			
c.137G>A p.(Cys46Tyr)	All BC	3000	3	0.1	NA	NA	0.95
rs746085217	*Familial BC*	1252	1	0.1	NA	NA	0.95
	Unselected BC	2347	2	0.1	NA	NA	0.96
	ER-positive BC	2296	3	0.1	NA	NA	0.94
	ER-negative BC	537	0	0.0	NA	NA	NA
RAD54L	Controls	2064	87	4.2			
c.1759C>T p.(Arg587Trp)	All BC	3001	145	4.8	1.18	0.90—1.56	0.24
rs150315374	*Familial BC*	1252	64	5.1	1.27	0.90—1.79	0.16
	Unselected BC	2348	114	4.9	1.18	0.88—1.57	0.27
	ER-positive BC	2297	113	4.9	1.21	0.91—1.62	0.20
	ER-negative BC	537	27	5.0	1.22	0.77—1.88	0.39
REC8	Controls	2062	18	0.9			
c.91C>T p.(Arg31Cys)	All BC	2997	39	1.3	1.61	0.92–2.91	0.10
rs34075659	*Familial BC*	1250	18	1.4	1.75	0.88–3.46	0.11
	Unselected BC	2347	28	1.2	1.46	0.81–2.70	0.22
	ER-positive BC	2295	28	1.2	1.45	0.80–2.71	0.23
	ER-negative BC	536	7	1.3	1.72	0.66–4.04	0.23
RECQL4	Controls	1269	5	0.4			
c.1390+2del [1]	All BC	2481	15	0.6	1.54	0.53–5.42	0.48
rs386833843	*Familial BC*	1136	6	0.5	1.34	0.34–5.57	0.77
	Unselected BC	1725	12	0.7	1.77	0.58–6.43	0.33
	ER-positive BC	1878	12	0.6	1.63	0.53–5.91	0.46
	ER-negative BC	452	1	0.2	0.56	0.01–5.03	1.00
RNASEL	Controls	2064	34	1.6			
c.793G>T p.(Glu265Ter)	All BC	3000	58	1.9	1.18	0.77–1.84	0.46
rs74315364	*Familial BC*	1253	23	1.8	1.11	0.63–1.90	0.72
	Unselected BC	2347	45	1.9	1.17	0.75–1.85	0.50
	ER-positive BC	2296	47	2.0	1.25	0.80–1.99	0.33
	ER-negative BC	538	9	1.7	1.01	0.45–2.05	0.98
SPHK1	Controls	2076	1	0.05			
c.35_36del	All BC	3146	7	0.2	4.13	0.72–77.9	0.19
p.(Phe12TrpfsTer46)	*Familial BC*	1368	4	0.3	5.98	0.84–120	0.12
rs746915055	*Unselected BC*	2390	4	0.2	3.30	0.48–64.8	0.29
	ER-positive BC	2387	5	0.2	4.04	0.64–78.4	0.21
	ER-negative BC	576	1	0.2	4.28	0.16–113	0.31
SPHK1	Controls	1271	4	0.3			
c.596del [1]	All BC	2479	9	0.4	1.15	0.32–5.14	1.00
p.(Arg199LeufsTer17)	*Familial BC*	1135	3	0.3	0.84	0.12–4.97	1.00
rs549579958	*Unselected BC*	1723	6	0.3	1.11	0.26–5.34	1.00
	ER-positive BC	1876	7	0.4	1.19	0.30–5.54	1.00
	ER-negative BC	452	2	0.4	1.41	0.13–9.86	0.66
STAP2	Controls	2066	37	1.8			
c.507C>G p.(Tyr169Ter)	All BC	2998	66	2.2	1.18	0.79–1.80	0.43
rs79657645	*Familial BC*	1249	31	2.5	1.39	0.85–2.29	0.19
	Unselected BC	2347	47	2.0	1.10	0.71–1.71	0.68
	ER-positive BC	2296	49	2.1	1.16	0.75–1.81	0.51
	ER-negative BC	536	13	2.4	1.37	0.69–2.56	0.33

Supplementary Table S2 continues

Variant	Series	Total	Carriers	%	OR	95% CI	P value
TMEM102	Controls	1268	16	1.3			
c.215-17_218del [1]	All BC	2387	32	1.3	1.06	0.56–2.08	0.88
rs537361079	*Familial BC*	1041	22	2.1	1.69	0.84–3.46	0.14
	Unselected BC	1725	16	0.9	0.73	0.34–1.57	0.47
	ER-positive BC	1819	23	1.3	1.00	0.50–2.04	1.00
	ER-negative BC	431	4	0.9	0.73	0.18–2.29	0.80
UVSSA	Controls	1270	0	0.0			
c.55C>T p.(Arg19Ter) [1]	All BC	2475	5	0.2	NA	NA	0.17
rs199574083	*Familial BC*	1136	3	0.3	NA	NA	0.11
	Unselected BC	1719	2	0.1	NA	NA	0.51
	ER-positive BC	1875	3	0.2	NA	NA	0.28
	ER-negative BC	449	0	0.0	NA	NA	NA

The variants denoted with [1] were analyzed with Fisher's exact test in the Helsinki breast cancer (BC) series only. The other variants were analyzed with region-adjusted logistic regression in the Helsinki and Tampere BC series. The familial and the unselected patient groups overlap with 380 individuals in the Helsinki series and with 614 individuals in the combined Helsinki and Tampere series.

Supplementary Table S4: Risk association analyses of additional cancer types from FinnGen for heterozygous *NTHL1* c.244C>T carriers

Cancer type		Total number of individuals		Additive model		
		Patients	Controls	OR	95% CI	P value
Gynecologic	Ovarian cancer	1091	182927	0.77	0.38–1.56	0.47
	Uterine cancer	2079	182927	0.97	0.59–1.59	0.90
	Cervical cancer	388	182927	0.74	0.23–2.43	0.62
Skin	Melanoma	3194	314193	0.98	0.65–1.49	0.94
	Non-melanoma skin cancer	19077	314193	1.15	0.96–1.39	0.14
	Squamous cell carcinoma of the skin	3531	314193	0.76	0.50–1.14	0.18
Endocrine	Pancreatic cancer	1626	314193	1.44	0.79–2.63	0.23
	Thyroid cancer	1906	314193	0.87	0.52–1.46	0.61
Hematologic	Acute myeloid leukaemia	244	314193	1.43	0.28–7.27	0.67
	Non-Hodgkin lymphoma	1072	314193	1.31	0.65–2.67	0.45
Other	Small intestine cancer	525	314193	0.98	0.37–2.63	0.97
	Brain cancer	816	314193	1.19	0.53–2.68	0.67
	Head and neck cancer	2281	314193	0.94	0.58–1.53	0.80
	Lung and bronchus cancer	6340	314193	0.97	0.71–1.31	0.83

The controls for ovarian, uterine, and cervical cancer included only women.

Supplementary Table S5: Cancer endpoints from FinnGen

Phenocode	Phenotype
C3_AML_EXALLC	Acute myeloid leukaemia
C3_BASAL_CELL_CARCINOMA_EXALLC	Basal cell carcinoma
C3_BLADDER_EXALLC	Malignant neoplasm of bladder
C3_BRAIN_EXALLC	Malignant neoplasm of brain
C3_BREAST_ERNEG_EXALLC	Malignant neoplasm of breast, ER-negative
C3_BREAST_ERPLUS_EXALLC	Malignant neoplasm of breast, ER-positive
C3_BREAST_EXALLC	Malignant neoplasm of breast
C3_BRONCHUS_LUNG_EXALLC	Malignant neoplasm of bronchus and lung
C3_CERVIX_UTERI_EXALLC	Malignant neoplasm of cervix uteri
C3_COLON_ADENO_EXALLC	Colon adenocarcinoma
C3_COLON_EXALLC	Malignant neoplasm of colon
C3_COLORECTAL_ADENO_EXALLC	Colorectal adenocarcinoma
C3_COLORECTAL_EXALLC	Colorectal cancer
C3_CORPUS_UTERI_EXALLC	Malignant neoplasm of corpus uteri
C3_HEAD_AND_NECK_EXALLC	Malignant neoplasm of head and neck
C3_MELANOMA_SKIN_EXALLC	Malignant melanoma of skin
C3_NONHODGKIN_EXALLC	Non-Hodgkin lymphoma
C3_OTHER_SKIN_EXALLC	Other malignant neoplasms of skin (=non-melanoma skin cancer)
C3_OVARY_EXALLC	Malignant neoplasm of ovary
C3_PANCREAS_EXALLC	Malignant neoplasm of pancreas
C3_PROSTATE_EXALLC	Malignant neoplasm of prostate
C3_RECTUM_ADENO_MUCINO_EXALLC	Adenocarcinoma, papilloma adenocarcinoma and mucinous carcinomas of rectum
C3_RECTUM_EXALLC	Malignant neoplasm of rectum
C3_RENAL_PELVIS_EXALLC	Malignant neoplasm of renal pelvis
C3_SMALL_INTESTINE_EXALLC	Malignant neoplasm of small intestine
C3_SQUOMOUS_CELL_CARCINOMA_SKIN_EXALLC	Squamous cell neoplasms and carcinomas of the skin
C3_THYROID_GLAND_EXALLC	Malignant neoplasm of thyroid gland
C3_URINARY_TRACT_EXALLC	Malignant neoplasm of urinary organs

Supplementary Information Methods: Genotyping

The Sequenom MassARRAY genotyping with iPLEX Gold assay (Sequenom, San Diego, CA, USA) was performed by the Genotyping laboratory of Institute for Molecular Medicine Finland Technology Centre (FIMM), University of Helsinki. The TaqMan genotyping was carried out using Custom TaqMan SNP Genotyping Assays (Thermo Fisher Scientific, Waltham, MA, USA), the PCR was run on 7500 Fast Real-Time PCR System or 9800 Fast Thermal Cycler, and 7500 Fast System SDS software v1.3.1 or 7500 software v2.0.6 (Applied Biosystems, Waltham, MA, USA) were used for genotype calling. Genotyping on 3% agarose gel electrophoresis was performed with short PCR amplicons of about 200 base pairs to separate the samples with indels (displaying two bands on gel) from the wildtypes (one band). The Sanger sequencing was done using BigDye Terminator v3.1 Cycle Sequencing kit (ThermoFisher Scientific) and the capillary electrophoresis was carried out at FIMM with ABI3730XL DNA Analyzer (Applied Biosystems). Positive samples confirmed by Sanger sequencing were used in gel electrophoresis and in TaqMan real-time PCR to control the quality of genotyping.

Biobank sample and data accession numbers for FinnGen

The Biobank Access Decisions for FinnGen samples and data utilized in the FinnGen data release 10 include: THL Biobank BB2017_55, BB2017_111, BB2018_19, BB_2018_34, BB_2018_67, BB2018_71, BB2019_7, BB2019_8, BB2019_26, BB2020_1, and BB2021_65; Finnish Red Cross Blood Service Biobank 7.12.2017; Helsinki Biobank HUS/359/2017, HUS/248/2020, and HUS/150/2022 §12, §13, §14, §15, §16, §17, §18, and §23; Auria Biobank AB17-5154 and amendment #1 (17th Aug 2020) and amendments BB_2021-0140, BB_2021-0156 (26th Aug 2021, 2nd Feb 2022), BB_2021-0169, BB_2021-0179, and BB_2021-0161, AB20-5926 and amendment #1 (23th April 2020) and its modification (22nd Sept 2021); Biobank Borealis of Northern Finland 2017_1013, 2021_5010, 2021_5018, 2021_5015, 2021_5023, 2021_5017, and 2022_6001; Biobank of Eastern Finland 1186/2018 and amendment 22§/2020, 53§/2021, 13§/2022, 14§/2022, and 15§/2022; Finnish Clinical Biobank Tampere MH0004 and amendments (21st Feb 2020, 6th Oct 2020) §8/2021, §9/2022, §10/2022, §12/2022, §20/2022, §21/2022, §22/2022, and §23/2022; Central Finland Biobank 1-2017 and Terveystalo Biobank STB 2018001 and amendment (25th Aug 2020); Finnish Hematological Registry and Clinical Biobank decision 18th June 2021; and Arctic Biobank P0844: ARC_2021_1001.

Ethics statement for FinnGen

Patients and control subjects in FinnGen provided informed consent for biobank research, based on the Finnish Biobank Act. Alternatively, separate research cohorts, collected prior the Finnish Biobank Act came into effect (in Sept 2013) and start of FinnGen (in Aug 2017), were collected based on study-specific consents and later transferred to the Finnish biobanks after approval by Fimea (Finnish Medicines Agency), the National Supervisory Authority for Welfare and Health. Recruitment protocols followed the biobank protocols approved by Fimea. The Coordinating Ethics Committee of the Hospital District of Helsinki and Uusimaa (HUS) statement number for the FinnGen study is Nr HUS/990/2017.

The FinnGen study is approved by Finnish Institute for Health and Welfare (permit numbers: THL/2031/6.02.00/2017, THL/1101/5.05.00/2017, THL/341/6.02.00/2018, THL/2222/6.02.00/2018, THL/283/6.02.00/2019, THL/1721/5.05.00/2019, and THL/1524/5.05.00/2020), Digital and Population Data Service Agency (permit numbers: VRK43431/2017-3, VRK/6909/2018-3, and VRK/4415/2019-3), the Social Insurance Institution (permit numbers: KELA 58/522/2017, KELA 131/522/2018, KELA 70/522/2019, KELA 98/522/2019, KELA 134/522/2019, KELA 138/522/2019, KELA 2/522/2020, and KELA 16/522/2020), Findata (permit numbers: THL/2364/14.02/2020, THL/4055/14.06.00/2020, THL/3433/14.06.00/2020, THL/4432/14.06/2020, THL/5189/14.06/2020, THL/5894/14.06.00/2020, THL/6619/14.06.00/2020, THL/209/14.06.00/2021, THL/688/14.06.00/2021, THL/1284/14.06.00/2021, THL/1965/14.06.00/2021, THL/5546/14.02.00/2020, THL/2658/14.06.00/2021, and THL/4235/14.06.00/2021), Statistics Finland (permit numbers: TK-53-1041-17, TK/143/07.03.00/2020 (earlier TK-53-90-20), TK/1735/07.03.00/2021, and TK/3112/07.03.00/2021), and Finnish Registry for Kidney Diseases (permission/extract from the meeting minutes on 4th July 2019).
